# Comparing the Immunomodulatory Properties of Bone Marrow, Adipose Tissue, and Birth-Associated Tissue Mesenchymal Stromal Cells

**DOI:** 10.3389/fimmu.2015.00560

**Published:** 2015-11-03

**Authors:** Philipp Mattar, Karen Bieback

**Affiliations:** ^1^Stem Cell Laboratory, Medical Faculty Mannheim, Institute of Transfusion Medicine and Immunology, Heidelberg University, Heidelberg, Germany; ^2^German Red Cross Blood Service Baden-Württemberg – Hessen, Mannheim, Germany

**Keywords:** mesenchymal stromal cells, immunomodulation, T cells, regulatory T cells, macrophages, bone marrow, adipose tissue, umbilical cord

## Abstract

Mesenchymal stromal cells (MSC) have gained immense attraction in regenerative medicine, tissue engineering, and immunotherapy. This is based on their differentiation potential and the supply of pro-regenerative and immunomodulatory signals. MSC can be isolated from a multitude of tissue sources, but mainly bone marrow, adipose tissue, and birth-associated tissues (e.g., umbilical cord, cord blood, placenta) appear to be relevant for clinical translation in immune-mediated disorders. However, only a few studies directly compared the immunomodulatory potency of MSC from different tissue sources. This review compiles the current literature regarding the similarities and differences between these three sources for MSCs with a special focus on their immunomodulatory effects on T-lymphocyte subsets and monocytes, macrophages, and dendritic cells.

## Introduction

Human mesenchymal stromal cells (MSC) have been an attractive target for translational research in a wide range of therapeutic applications due to their paracrine effects, multi-lineage differentiation potential and, most importantly, their immunomodulatory properties (1, 2). Bone marrow was the first established source of these cells (3). Since then it has been the primary and therefore most investigated population. Over the last few years, several other sources for MSC have been identified (4, 5). In our view, after bone marrow-derived MSCs (hereafter referred to as BM-MSC), adipose tissue-derived MSCs (hereafter referred to as AT-MSC), and birth-associated MSCs (from umbilical cord blood, cord tissue, and placenta, referred to as UCB-MSC, UC-MSC, and PL-MSC, respectively) (6) are most commonly used as sources of human MSCs in a clinical setting. These may be favored due to different advantages: greater yields of MSCs than BM, higher proliferative potential, and no ethical restrictions (5–10). It has to be noted that birth-associated tissue is not one single source of MSCs but rather a comprising name for several subpopulations, namely amnion, umbilical cord, cord blood, and placenta. Most of them can be categorized further into even more subpopulations.

Mesenchymal stromal cells from different sources are similar in a range of phenotypic and functional features (5). There are, however, subtle differences, which may result from the microenvironmental niche, the local function (stromal support of hematopoiesis in the BM and immune homeostasis by AT), and the ontogenetic age (birth-associated versus adult) (5, 7, 11) or induced by the isolation and culture procedure. CD106 is one example, as it is significantly reduced on AT-MSCs compared to other MSCs (5, 12, 13). CD34, on the other hand, appears on AT-MSCs *in situ* and early in culture but on no other MSCs (14, 15). Regarding function, we and others reported for example significantly reduced adipogenic differentiation capacity of UCB-MSC (16). Regarding the stromal supportive capacity, a recent study indicates that only BM-MSC (not MSC from white adipose tissue, umbilical cord, and skin) are capable to form a functional hematopoietic niche (17).

Immunomodulatory functions have been reported for all types of MSC tested. Strikingly, analyses directly comparing these populations with their immunomodulatory effects are limited. As many scientific groups just use one single source for MSCs in their experiments – indeed beneficial for the reproducibility of their own data – it renders it hard to compare the results to those of other scientists and to draw conclusions about their clinical efficacy. To assess immunomodulation, most groups utilize a mixed lymphocyte reaction (MLR) assay or an assay measuring T cell proliferation induced by mitogens or CD3/CD28 stimulation. Fewer groups address distinct effects on T cell subsets (Th1, Th2, Th17, and regulatory T cells) and antigen-presenting cells (APCs) [reviewed in Ref. (2, 18, 19)]. Although the vast majority of studies confirm MSCs to inhibit the immune response, recent data identified allogeneic MSCs to be immunogenic and immune-rejected under appropriate conditions (20–22). There is a large diversity in soluble factors to mediate the effects of MSCs, thus it remains to be clarified whether MSC origin and culture conditions use different molecular mechanisms to exert their effects (2, 23). Some interesting data suggest intrinsic differences in expression of immune-related signature genes, mi- and tRNA species (24, 25). However, a summary of these is beyond the scope of this review. Here, we focused on studies, which directly compared two or more MSC tissue sources addressing MSC effects on T cell subpopulations or APCs, such as monocytes, macrophages, or dendritic cells (DCs) (summarized in Table 1).

**Table 1:** **Studies directly comparing different sources of MSCs, reporting differences in immunomodulatory capacities**.

Reference	Populations compared	Parameters	Outcome
Bárcia et al. (26)	BM, UC	Immunomod^a^	UC > BM
		Immunogenicity	UC < BM
Barlow et al. (9)	BM, PL	Proliferation	PL > BM
		Immunogenicity	BM = PL
Castro-Manrreza et al. (27)	BM, UCB, PL	Immunomod^b^	BM = UCB > PL
Hass et al. (7)	BM, AT, UC	Proliferation	UC > AT > BM
		Senescence	UC < AT < BM
Ivanova-Todorova et al. (28)	BM, AT	Immunomod^c^	AT > BM
Jin et al. (29)	BM, AT, UCB	Proliferation	UCB > AT = BM
		Immunomod^d^	UCB > AT = BM
		Senescence	UCB < AT = BM
Kern et al. (5)	BM, AT, UCB	Proliferation	UCB > AT > BM
		Isolation success rate	BM = AT > UCB
		Colony frequency	AT > BM > UCB
Li et al. (30)	BM, AT, UC, PL	Proliferation	WJ > AT > PL > BM
		Immunomod^e^	WJ > PL > AT > BM
Luan et al. (31)	BM, PL	Immunomod^f^	BM = PL
Montespan et al. (32)	BM, AT	Immunomod^g^	AT > BM
Najar et al. (33, 34)	BM, AT, UC	Immunomod^h^	AT > BM = UC
Prasanna et al. (22)	BM, WJ	Immunogenicity	BM = WJ
		Immunomod^i^	WJ ≠ BM
Puissant et al. (35)	BM, AT	Immunogenicity	BM = AT
		Immunomod^j^	BM = AT
Ribeiro et al. (36)	BM, AT, UC	T/NK cell inhibition	AT > BM = UC
		B cell inhibition	BM = AT (UC none)
Roemeling-van Rhijn et al. (37, 38)	BM, AT	Immunomod^k^	BM = AT
		Immunomod^l^	AT < BM
Stubbendorf et al. (39)	UCB, WJ, PL, UCL	Proliferation	UCL > UCB > WJ = PL
		Immunomod^m^	UCL > UCB = WJ = PL
		Immunogenicity	UCL ≤ PL ≤ WJ = UCB
Xishan et al. (12)	BM, AT	Proliferation	AT > BM
		Immunomod^n^	BM > AT
Yoo et al. (40)	BM, AT, UCB, WJ	Immunomod^f^	BM = AT = UCB = WJ
		Cytokines^o^	Only UCB and WJ

*^a^MSCs + PBMCs/T cells. MLR assay to assess lymphocyte proliferation and immunogenicity. Flow cytometry to measure Treg induction. Comparative gene expression analysis*.

*^b^MSCs + T cells (±transwell). Proliferation assay for CD4^+^ and CD8^+^ T cells. Flow cytometry to assess T cell activation and CTLA-4 and PD-L1 expression. Multiplex assay to measure IFN-γ, TNF-α, IL-10, and IL-4*.

*^c^MSCs + Monocytes. Flow cytometry to assess CD14, CD80, CD83, CD86, and HLA-DR. ELISA to measure IL-10 and IL-18. Proteome profile assay for 36 cytokines (e.g., CCL-3 and CCL-4)*.

*^d^MSCs + LPS stimulated rat macrophages. ELISA to assess IL-1α, IL-6, and IL-8 and Ang-1*.

*^e^MSCs + T cells. T cell proliferation was assessed*.

*^f^MSCs + T cells. T cell proliferation assay. ELISA to assess IFN-γ and IL-10, or TNF-α*.

*^g^MSCs + PBMCs. Flow cytometry analysis for HLA-G. MLR assay to assess immunosuppression*.

*^h^MSCs + mitogenic/allogenic stimulated T cells. T cell activation and proliferation assays. Subset analysis for CD4^+^ and CD8^+^ T cells. PCR for COX1 and COX2. ELISA for PGE2 MSCs + T cells. MSCs were primed with IFN-α, IFN-γ, TNF-α, or IL-1β or unstimulated. T cell proliferation assay. Flow cytometry to assess lymphocyte activation. ELISA for IFN-γ, IL-8, and CCL5. T cell migration assay*.

*^i^MSC + PBMCs stimulated with PHA or MLR; MSC primed with IFN-γ or TNF-α: immunogenicity and T cell proliferation; PBMC cytokine profiles, activation markers, and immune-suppressive factors (IDO, PGE2, HGF, CIITA)*.

*^j^MSCs + PBMCs: MLR or mitogen-induced T cell proliferation, time- and dose-dependent suppression, dependent on soluble mediators (but most probably not TGF-β, HGF, and IL-10)*.

*^k^MSCs + PBMCs: PBMC proliferation assay. PCR for IDO, TGF-β, and CXCL-10. Application of PBMCs and MSCs in an *in vivo* mouse allograft rejection model*.

*^l^MSCs and CD8^+^ T cells: induction of HLA-specific alloreactivity by MSC-educated CD8^+^ TC*.

*^m^MSCs stimulated with IFN-γ and MSCs + T cells. ELISA for IL-2, IL-10, and TGF-β1. Electrophoresis for IDO*.

*^n^MSCs + PHA stimulated T cells. Effects on T cell proliferation, MLR assay, T cell cycle, T cell apoptosis, early activation, and T cell subsets were assessed*.

*^o^MSC + PHA-stimulated T cells: cytokines: IL-12, IL-15, and PDGF-AA*.

*AT, adipose tissue; BM, bone marrow; MLR, mixed lymphocyte reaction; MSC, mesenchymal stromal cells; PBMC, peripheral blood mononuclear cells; PL, placenta; Treg, regulatory T cells; CTL, cytotoxic T lymphocytes; DC, dendritic cells UC, umbilical cord; UCB, umbilical cord blood; WJ, Wharton’s jelly*.

## Effects on T Cells

### Effects on Naïve CD4^+^ T Cells

The exerted effects on naïve CD4^+^ T cells are of a suppressing and polarizing nature, meaning MSCs inhibit the proliferation and activation of naïve CD4^+^ T helper cells (Th cells). They are able to influence the differentiation of Th0 cells into Th1, Th2, Th17, or regulatory T cells (Tregs) (36, 41, 42). MSCs seem to hamper T cell proliferation by arresting T cells in the G0/G1 phase of the cell cycle (12, 43), thus reducing the total number of T cells undergoing activation. MSCs exert their immunomodulatory functions through numerous molecules. Although trans-well experiments show an inhibiting function of MSCs, most studies confirm a more pronounced effect without trans-wells, highlighting the importance of cell-cell contact in mediating immunomodulatory functions. Prostaglandin E2 (PGE2) seems to play an important role in suppressing the immune response (33). Just recently, evidence arose that MSC-derived microvesicles contain a variety of immunomodulatory factors, including miRNA and tRNA species (25, 44). Di Nicola et al. proposed transforming growth factor (TGF)-β and hepatocyte growth factor (HGF) as important mediators, as blockage of both significantly reduced the suppressive effect of MSCs (45). Another group identified indoleamine 2,3-dioxygenase (IDO) to be involved (46). IDO catalyzes the conversion of tryptophan, an essential molecule in the activation of T cells, to kynurenine and has been identified as a key pathway for inhibiting T cell response. Additionally, Human Leukocyte Antigen-G5 (HLA-G5) was found to be required to suppress T cell function and to induce Tregs (32, 47).

#### Comparison

Comparative studies have produced conflicting results. Puissant et al. report similar inhibition of T cell proliferation, both induced in MLR or mitogens, in presence of BM- or AT-MSCs (35). In both settings suppression was induced by soluble mediators. In contrast, whereas Ribeiro et al. (36) found AT-MSCs (compared to BM-MSCs and UC-MSCs) to have the strongest suppressive effect on the activation and acquisition of lymphoblast characteristics on T cells, Xishan et al. (12) determined BM-MSCs to have a superior immunosuppressive effect over AT-MSCs. In a study comparing MSCs from bone marrow, adipose tissue and Wharton’s jelly, AT-MSCs showed the strongest effect on downregulating the activation marker CD38 on T cells, followed by UC-MSCs, whereas BM-MSCs had the weakest effect (33). The authors showed AT-MSCs to be the most potent population in inhibiting allogeneic-induced T cell proliferation (33). Interestingly, different levels of the enzyme cyclooxygenase-1 (COX1), which is essential in PGE2 production, were observed, with highest levels of COX1 in AT-MSC (33). Conflicting data, however, was presented by Li et al., who determined MSCs from Wharton’s jelly to possess the strongest inhibitory effect on T cell proliferation compared to AT-MSCs, BM-MSCs, and PL-MSCs (30). Similar inhibitory effects on T cell proliferation, activation, and cytokine secretion are reported by Luan et al. comparing PL- and BM-MSC (31). They identified programed death-ligand 1 (PD-L1, CD274, or B7-H1) and B7H4 as negative regulators.

### Effects on CD4^+^ Th1 Cells

A large number of studies have been performed to explore the effects of MSCs on Th1 cells, considered to be the main effector cells of proinflammatory cell-mediated immunity and organ-specific autoimmune disorders (23, 48, 49). The results obtained from these studies usually imply an inhibiting effect on Th1 cells (12, 41, 48–50). However, there are conditions in which MSCs seem to promote Th1 cells and inhibit the differentiation of Th2 cells (49). Cho et al. described AT-MSCs to reduce Th2-associated cytokines (interleukin IL-4, IL-5) and increase Th1-derived interferon (IFN)-γ and IL-2 in a model of eosinophilic nasal polyps (51). These data are corroborated by other studies, confirming the Th2-inhibiting function of MSCs in Th2-dominated inflammatory conditions, such as allergic airway inflammation (52). In an inflammatory environment, high levels of IFN-γ and/or tumor necrosis factor (TNF)-α increase the expression of TGF-β by MSCs (53, 54), which in turn prompts Th1 cells to express immunosuppressive IL-10 and ultimately reduces their IFN-γ production. Furthermore, MSC mediate a downregulation of the Th1 cells IFN-γ receptor, which renders them less susceptible to IFN-γ (55). In a recent study, MSCs-induced and expanded a subpopulation of T-bet^+^ Th1 cells co-expressing IFN-γ and IL-10 (55). T-bet is a Th1 cell-specific transcription factor (56). This suggests that the influence of MSCs on IFN-γ expression is dependent on several factors, such as the cytokine milieu, the stimulation methods or the types of cells present, showing that we are far from having a full grasp of the effects of MSCs on immune cells.

#### Comparison

AT-MSCs and BM-MSCs showed similar results in inhibiting Th1 differentiation, as both significantly reduced the levels of IL-2 and IFN-γ (12). Another study compared several MSC populations from birth-associated tissue (umbilical cord lining, cord blood, placenta, and Wharton’s jelly), resulting in cord lining MSCs to emerge as the most potent in dampening Th1 and Th2 responses and reducing release of IFN-γ by lymphocytes (39). Castro-Manrreza et al. compared BM-MSCs, UC-MSCs, and PL-MSCs and identified similar proliferation suppression capacities for BM-MSCs and UC-MSCs, but PL-MSCs showed significantly weaker CD4^+^/CD8^+^ T lymphocyte suppression (27). AT-MSCs exerted the strongest inhibition on IFN-γ secretion and T cell proliferation compared to BM-MSCs and WJ-MSCs (34).

### Effects on CD4^+^ Th2 Cells

Th2 cells have several functions in the humoral-mediated immune response, as they host the defense against extracellular parasites, inhibit Th1 cells and DCs via IL-10, stimulate B cells via IL-4 and can induce isotype-switches in B cells (57). MSCs have been shown to enhance anti-inflammatory IL-4 production by Th2 cells, supposedly via a PGE2 (48). In inflammatory diseases that are associated with high amounts of Th2 cells (e.g., allergies, asthma, Crohn’s disease), MSCs were able to ameliorate disease activity by inhibiting the cytokine production of Th2 cells (IL-4 and IL-5) and increase Th1-derived cytokines (IFN-γ and IL-2) (51, 58).

#### Comparison

There are a small number of studies concentrating on the comparison of various MSC sources on T cell subsets. Xishan et al. compared AT-MSCs and BM-MSCs on their ability to induce Th0 differentiation into Th1 and Th2 cells and could show that both populations had no significant effect on the levels of the Th2-associated cytokines IL-4 and IL-10 (12). Concerning Th2 cells, data is especially scarce.

### Effects on CD4^+^ Th17 Cells

Th17 cells play an important role in the human immune system as effectors against extracellular bacterial and fungal infections, but have also been associated with autoimmune diseases, such as multiple sclerosis, psoriasis, rheumatoid arthritis, inflammatory bowel disease, systemic lupus erythematosus, and asthma (59). Although studies about the effects of MSCs on Th17 cells seem to yield rather consistent results, presenting MSCs as potent inhibitors of Th17-mediated immune responses (60–62), data exists where Th17 cells appear to be stimulated by MSCs *in vitro* (63). The time at which MSCs are added could be important, as Carrión et al. demonstrated opposing effects of MSCs on Th1 and Th17 cells relative to the state of CD4^+^ T cell activation (49).

#### Comparison

AT-MSCs, UC-MSCs, and BM-MSCs have all proven to be effective in suppressing the Th17 immune response (41, 60, 64), but studies directly comparing them are rare. In a mouse model of experimental colitis, UC-MSCs and BM-MSCs demonstrated a similar inhibition of Th17 cells, shifting the Th17/Treg ratio toward a more immunosuppressive balance (64).

### Effects on CD4^+^ Foxp3+ Regulatory T Cells (Tregs)

Regulatory T cells are either derived in the thymus as mature Tregs, or from CD4^+^CD25^−^ naïve T cells as peripherally derived Tregs under the influence of TGF-β and IL-2 (65). Tregs target effector T cells and DCs (65, 66) by inhibiting their differentiation, function, and maturation to prevent autoimmunity and establish a peripheral tolerance (67). MSCs have been shown to induce Tregs via a multitude of factors. HLA-G5, a non-classical HLA class I molecule, plays an important role in the induction of Tregs (68). Another factor of MSCs involved in the activation of Tregs is TGF-β, which seems to be constitutively expressed by MSCs (69). Additionally, MSCs were reported to elevate IL-10 production by Tregs and DCs (70, 71), whereby DC-derived IL-10 in turn promotes the expansion of Tregs (72). Tregs can also be indirectly activated by MSCs through an upregulation of Fas ligand (FasL)/Fas-mediated death pathway, which targets T cells via cell-cell contact and leads to increased apoptosis and Treg induction (73). In several *in vivo* settings, MSCs increased Tregs, thereby ameliorating disease states as well as promoting graft survival in transplant experiments (41, 50, 74–76).

#### Comparison

In an *in vitro* study that compared BM-MSCs and UC-MSCs on their ability to induce Tregs, UC-MSCs had a significantly greater potential to induce Tregs than BM-MSCs (26). Chao et al., on the other hand, did not report a difference in Treg induction of BM-MSCs and UC-MSCs in an *in vivo* experiment (77).

### Effects on CD8^+^ T Cells (CTL)

Cytotoxic T lymphocytes (CTLs) are major effectors in the immune system through targeting virus-infected cells as well as tumor cells. CTLs have a crucial role in autoimmunity and transplant rejection. CTL activation is triggered following the interaction of the T cell receptor (TCR) with the specific allogeneic peptide–HLA-I complex. The activation of lymphocytes can be divided into several steps, which all have a corresponding phenotype: CD69^−^CD25^−^HLA-DR^−^ (non-activated), CD69^+^CD25^−^HLA-DR^−^ (earlier activated), CD69^+^CD25^+^HLA-DR^−^ (intermediate activated) and CD69^+^CD25^+^HLA-DR^+^ (later activated). It was reported that MSCs are able to dampen the immune response of CTLs as well as inhibiting their proliferation and maturation (36, 37, 51). MSC downregulate the CD8 surface marker on CTLs via an indirect pathway involving CD14^+^ monocytes, requiring cell-cell contact between the monocytes and the CTLs (78). In this process, CD28 is downregulated on CTLs indicating loss of effector-type and gain of regulatory functions (78).

#### Comparison

Ribeiro et al. investigated AT-MSCs, BM-MSCs, and UC-MSCs as to their effect of inhibiting CD4^+^/CD8^+^ lymphocyte activation (36). Co-culture with BM-MSCs and UC-MSCs similarly inhibited lymphocyte activation, whereas the majority of the CD8^+^ cells were of the earlier activated phenotype. AT-MSCs here emerged as the most immunosuppressive population, as the majority of the T Cells were found to be in the non-activated compartment (36). Different effects on CD8^+^ mediated alloreactivity are reported by Roemeling-van Rhijn et al., addressing the capacity of AT- versus BM-MSC to induce HLA-specific alloreactivity (38). CD8^+^ T cells educated with IFN-γ-treated AT-MSC evoked 31% specific lysis of AT-MSCs with identical HLA. IFN-γ-treated BM-MSC, however, resulted in 76% HLA-specific killing of HLA-identical BM-MSC.

## Effects on Monocytes, Macrophages, and Dendritic Cells

Monocytes are a subpopulation of leukocytes able to differentiate into macrophages and DCs. Macrophages and DCs are antigen-presenting cells that can initiate an immune response and act as a mediator between the innate and the adaptive immune system. MSCs were reported to strongly induce the secretion of IL-10 on CD14^+^ monocytes via HGF, thereby suppressing T cell proliferation (79). Melief et al. could show that MSCs promote the survival of monocytes and induce the differentiation into CD163^+^ CD206^+^ type 2 macrophages, which secrete IL-10 and CCL18 (69). CCL18 in turn has a crucial role in inducing Tregs (69). MSCs were frequently shown to be able to inhibit the proinflammatory functions of DCs and macrophages and skew the cells toward a more immunosuppressive response (70, 71, 80). The proinflammatory molecules TNF-α and macrophage inflammatory protein (MIP)-1β, produced by macrophages and mature DCs, were suppressed under the influence of MSCs (80). Concerning maturation markers such as CD1a, CD14, CD83 and HLA-DR, MSCs inhibited the maturation of DCs and furthermore downregulated the costimulatory molecules CD86/CD80 (81, 82). Conflicting data exist, as Laranjera et al. could not detect any influence of MSCs on maturation markers CD83, CCR7, and HLA-DR (80) on DCs, thus leading the group to suppose that MSCs exhibit their anti-inflammatory functions on macrophages and DCs mainly by inhibiting the secretion of proinflammatory cytokines. MSCs are also able to inhibit the differentiation at a more upstream step by interfering with monocyte maturation (83).

### Comparison

AT-MSCs seem to have a more pronounced effect on DC differentiation than BM-MSCs (28). Saeidi et al. examined UC-MSCs on their potential to interfere with maturation and endocytotic capability of DCs comparing them with BM-MSCs (81). While being equally effective in hampering the maturation of DCs, UC-MSCs had a stronger effect on reducing the endocytotic ability of DCs (81). Jin et al. compared anti-inflammatory activity of BM-, AT-, and UCB-MSCs (29). UCB-MSCs were most potent in suppressing cytokine release from LPS-challenged alveolar macrophages. Angiopoietin-1 was at least partly responsible for this effect.

## Conclusion

The increasing numbers of studies conducted on comparing MSC sources *in vitro* and *in vivo* yield largely congruent results, presenting MSCs as promising cells for a multitude of immunological applications (Table 1). Nevertheless, the heterogeneity in MSC populations and experimental protocols still poses a major obstacle when trying to compare and merge different results and to translate them into clinical practice (84). Our survey reflects that the vast majority of data showed no significant deficiencies in the immunomodulatory potential of MSCs from alternative sources but often even stronger immunosuppressive capabilities than BM-MSCs (26, 28, 30, 33, 34, 36, 81). This was especially the case for MSCs from adipose tissue (28, 32–34, 36). This claim, however, is based on the few studies directly comparing MSCs from different tissue sources. Future studies should elucidate whether the similarities of tissue MSCs *in vitro* relate to similar functions *in situ* or are artificially gained by *ex vivo* isolation and culture adaptation (85, 86) and whether the subtle differences in function relate to the role of MSCs *in situ*. What is certain is that MSCs expanded *in vitro* are highly sensitive to their microenvironment; they may alternate their function based on the surrounding conditions. Important parameters are the culture conditions (e.g., choice of serum supplement), types of immune cells present, cell activation status, ratio of MSC to immune cells and, of course, the cytokine levels in the milieu (87–90). Additionally, variations in isolation methods, culture media, cell counts, and different stimulation protocols can further blur the potential differences among distinct MSC sources. A standardization of assays to assess the effects of MSCs is essential to guarantee trustworthy and reproducible results (18). Ideally, these assays are capable of predicting efficacy of MSCs *in vivo*, to serve as potency assay. We would therefore appreciate more comparative studies to give us a better understanding of the immunomodulatory mechanisms of MSCs, facilitating the choice between different sources for defined clinical settings to improve safety and efficacy of MSC-based therapies.

## Author Contributions

PM, KB: conception, acquisition, analysis, or interpretation of data, drafting and critical revision, final approval before submission, and agreement to be accountable for all aspects of the work in ensuring that questions related to the accuracy or integrity of any part of the work are appropriately investigated and resolved.

## Conflict of Interest Statement

The authors declare that the work was conducted in the absence of any commercial or financial relationships that could be construed as a potential conflict of interest.

## Abbreviations

AT, adipose tissue; BM, bone marrow; CTL, cytotoxic T lymphocytes; DC, dendritic cells; MLR, mixed lymphocyte reaction; MSC, mesenchymal stromal cells; PBMC, peripheral blood mononuclear cells; PL, placenta; Treg, regulatory T cells; UC, umbilical cord; UCB, umbilical cord blood; WJ, Wharton’s jelly.
